# Treatment of compulsive exercise in eating disorders and muscle dysmorphia: protocol for a systematic review

**DOI:** 10.1186/s40337-021-00375-y

**Published:** 2021-02-10

**Authors:** Jordan Andre Martenstyn, Stephen Touyz, Sarah Maguire

**Affiliations:** 1 Clinical Psychology Unit, School of Psychology, The University of Sydney, Sydney, Australia; 2 InsideOut Institute for Eating Disorders, The Boden Collaboration for Obesity, Nutrition, Exercise and Eating Disorders, The University of Sydney, Sydney, Australia; 3 Sydney Local Health District, NSW Health, St Leonards, Australia

**Keywords:** Compulsive exercise, Exercise addiction, Eating disorders, Muscle dysmorphia, Bigorexia, Treatment, Intervention

## Abstract

**Background:**

Compulsive exercise is a core feature of both eating disorders and muscle dysmorphia. Earlier models of treatment recommended complete abstinence from exercise in eating disorder populations, but recent guidelines advocate for the gradual inclusion of healthier forms of exercise into an overall treatment plan where appropriate. Given the association between problematic exercise behaviour and poorer prognosis, there has been a recent upsurge in the number of treatment interventions for compulsive exercise in eating disorders. However, no systematic review has been published summarising this existing treatment literature. The aim of this review is to determine the efficacy of existing treatments for compulsive exercise in eating disorders and muscle dysmorphia.

**Methods:**

A systematic review will be conducted according to the Preferred Reporting Items for Systematic Reviews and Meta-Analyses (PRISMA) guidelines. Five electronic databases (PsycInfo, MEDLINE, Embase, Web of Science, and Scopus) will be searched from database inception until November 2020. We will include studies that: (a) sampled adolescents and/or adults with either an eating disorder or muscle dysmorphia; (b) assessed changes in compulsive exercise from pre- to post-intervention; and (c) used a standardised instrument to measure compulsive exercise or related constructs. We will include studies with a comparison group (e.g., randomised controlled trials) and without a comparison group (e.g., pilot studies and case studies) to provide a comprehensive overview of the literature. One reviewer will screen all titles and abstracts against eligibility criteria, with 20% of excluded articles cross-referenced by another reviewer. Full texts will be obtained for articles deemed relevant or where inclusion was uncertain, and will be screened by both reviewers. We will also evaluate the quality of the included studies using a modified Downs and Black (J Epidemiol Community Health 52:377–384, 1998) assessment checklist.

**Discussion:**

Results from this review will help to determine the most efficacious treatment components for compulsive exercise in eating disorders and muscle dysmorphia. We hope that our results will help inform clinical practice guidelines in recommending targeted interventions for the treatment of compulsive exercise.

## Background

Excessive exercise has long been considered an important feature of eating disorder psychopathology, mentioned in the earliest recorded medical description of anorexia nervosa [20]. In people with eating disorders, unhealthy exercise is associated with poorer prognosis, including lower quality of life [52], longer inpatient hospitalisations [46], greater suicidal behaviour [45], and increased risk of relapse [6]. There is growing agreement that unhealthy exercise and related constructs are best defined based on one’s qualitative relationship with exercise, rather than the quantitative amount of exercise performed, as what constitutes ‘excessive’ exercise will differ depending on age, fitness, and health status [8, 37]. However, to date, there is no consensus on what constitutes problematic exercise in the context of eating disorders [37]. Indeed, an array of terminology has been used to describe the unhealthy relationship with exercise often observed in people with eating disorders, such as compulsive exercise [4, 11, 25], exercise addiction [1, 17, 51], exercise dependence [3, 22], and obligatory exercise [47]. While these terms are often used interchangeably, they reflect different conceptualisations of problematic exercise behaviour: either as a behavioural addiction like gambling (exercise addiction), a dependence to exercise similar in nature to a substance dependence disorder (exercise dependence), or a behaviour that one feels obligated to perform, even in the absence of a pleasurable reward (obligatory exercise). A recent Delphi study indicated that 60% of international experts in the treatment of eating disorders preferred the term *compulsive exercise*, although it must be noted that 60% agreement did not meet their threshold for consensus (≥ 85% agreement) or near consensus (≥ 75% agreement [37]). Results from this Delphi study are consistent with other research positing that compulsive exercise offers the most theoretically sound construct to define problematic exercise in the context of eating disorders [27, 29, 43].

Compulsive exercise describes a highly driven and rigid urge to exercise, combined with a perceived inability to stop exercising, despite awareness of the risk of harm from continued exercise [49]. Estimates of the prevalence of compulsive exercise range from 16.7 to 85.3% in adolescents with eating disorders [16] and 31.9 to 80.0% in adults with eating disorders [9, 44]. Given this high crossover, the most recent clinical practice guidelines on eating disorders published by The Royal Australian and New Zealand College of Psychiatrists emphasised the importance of screening for compulsive exercise when assessing someone with a suspected eating disorder [23].

Earlier models of treatment recommended complete abstinence from exercise in underweight women for which weight restoration was the main treatment goal [21]. However, several reviews have demonstrated that performing structured exercise under supervision does not interfere with weight restoration in women with anorexia nervosa [30, 36, 53]. In fact, some exercise interventions in women with anorexia nervosa led to greater weight gain compared with those who did not exercise [5, 48]. In light of this evidence, current practice adopts a more nuanced approach, advocating for the gradual reintroduction of safe exercise into an eating disorder treatment program where appropriate [7, 10, 37].

Unhealthy exercise is also a core feature of muscle dysmorphia, an illness characterised by an intense preoccupation that one is not big enough or sufficiently muscular, despite often being significantly larger and more muscular than the average person [40, 41]. Although this phenomenon has been recognised in bodybuilding communities under the term *bigorexia* since the 1980s, it did not receive scientific attention until Pope et al. [41] identified nine bodybuilders (out of a larger sample of 108 bodybuilders) who had irrational beliefs that they were too small. Given this psychiatric presentation, the disorder was aptly labelled reverse anorexia [41]. However, it was later renamed as muscle dysmorphia and a set of diagnostic criteria was proposed that placed the compulsive need to exercise at the crux of muscle dysmorphia symptomology [40]. Although the literature on muscle dysmorphia is still in its infancy, it was recently included in the fifth edition of the Diagnostic and Statistical Manual of Mental Disorders as a specifier of body dysmorphic disorder [2], which has helped to spur additional research.

Compulsive exercise has been highlighted in the original diagnostic criteria for muscle dysmorphia [40] and a number of recent case studies on people with the disorder [31, 32]. This centrality of compulsive exercise may explain why the majority of studies on muscle dysmorphia have sampled adult male bodybuilders (e.g., [14, 26, 28, 38]). Murray et al. [33] found that men with muscle dysmorphia reported significantly greater compulsive exercise (*d* = 2.6) than male gym-goers without muscle dysmorphia and similar scores on three subscales of the Compulsive Exercise Test (avoidance and rule-driven behaviour, exercise rigidity, and mood improvement) when compared against men with anorexia nervosa. Men with muscle dysmorphia were also found to share other common features with men suffering from anorexia nervosa, including comparable weight and shape concern, appearance intolerance, and functional impairment [33]. However, it is unclear whether these concerns are similar in magnitude between men with muscle dysmorphia and women with anorexia nervosa. There is also evidence that disordered eating confers a risk factor for the development of muscle dysmorphia; Pope et al. [40] found that 22% of men with muscle dysmorphia previously met diagnostic criteria for anorexia nervosa, while Olivardia et al. [38] reported that 29% of men with muscle dysmorphia had a past diagnosis of any eating disorder. Given this intersection between muscle dysmorphia and eating disorders, some authors have argued that muscle dysmorphia should be nosologically classified as an eating disorder that captures the drive for muscularity more frequently associated with males [34, 35].

Although men with muscle dysmorphia are often admired for their self-control in maintaining strict exercise and diet practices [18], there is more to this disorder than meets the eye. Men with muscle dysmorphia report quality of life scores that are 1.7–2.6 standard deviations below community norms, indicating significant impairment to quality of life [39]. This same study also found that men with muscle dysmorphia are almost three times as likely to have attempted suicide compared to men with body dysmorphic disorder unrelated to muscularity [39], highlighting the importance of evidence-based treatment approaches.

In the past few years, there has been a rapid upsurge in the number of treatment interventions for compulsive exercise in eating disorders (e.g., [12, 24]). The majority of these studies sampled people with diagnosed anorexia nervosa (AN), bulimia nervosa (BN), eating disorder not otherwise specified (EDNOS; DSM-IV), or other specified feeding or eating disorder (OSFED; DSM-V). However, to our knowledge, no systematic review has been published summarising the existing treatment literature in eating disorder samples. One such review is urgently needed to identify the common treatment components (e.g., psychoeducation, cognitive challenging, sport therapy) driving effects across existing interventions. As compulsive exercise is also a defining characteristic of muscle dysmorphia [40], and this disorder shares many features with other eating disorders, specifically anorexia nervosa, suggesting that effective treatment components may be similar across the groups, we decided to make both eating disorders *and* muscle dysmorphia the focus of this review. Although significantly less treatment research has been conducted on muscle dysmorphia samples compared to eating disorder samples [19, 50], there is still scope to explore whether insight can be gained from the extant literature into the treatment of compulsive exercise in muscle dysmorphia.

The primary aim of this systematic review is to determine the efficacy of existing treatments for compulsive exercise in eating disorders and muscle dysmorphia. A secondary aim is to evaluate the methodological quality of included studies. We hope that findings from this review will inform evidence-based guidelines for the treatment of compulsive exercise in disorders defined by maladaptive preoccupations with body image.

## Methods

### Eligibility criteria

Our systematic review will be conducted according to the Preferred Reporting Items for Systematic Reviews and Meta-Analyses (PRISMA) guidelines and studies will be selected for inclusion in this review according to the criteria outlined below.

#### Study designs

Eligible studies must assess changes in compulsive exercise from pre- to post-intervention. This comprises studies that include a comparison group (e.g., randomised controlled trials) and those that do not include a comparison group (e.g., pilot studies and case studies). If data are reported for the same intervention across multiple studies, for example, a pilot study and a subsequent randomised controlled trial, only data from the randomised controlled trial will be extracted for narrative synthesis. Literature reviews, theoretical articles, and conference abstracts will be excluded.

#### Participants

We will include studies that sampled adolescents, defined as age above 10 years [42], and/or adult participants of both sexes who have been diagnosed with either an eating disorder or muscle dysmorphia. These clinical diagnoses must be determined using established diagnostic criteria, such as the Diagnostic and Statistical Manual of Mental Disorders (DSM) or the International Classification of Diseases (ICD). The list of possible eating disorder diagnoses includes AN, BN, binge eating disorder, EDNOS (DSM-IV), and OSFED (DSM-V). Although muscle dysmorphia is included in the DSM-V as a specifier of body dysmorphic disorder [2], it was not officially included in earlier versions of the DSM or any version of the ICD. Thus, we will also include studies carried out prior to publication of the DSM-V that used the diagnostic criteria for muscle dysmorphia outlined in Pope et al. [40]. Studies that used clinical cut-off scores from questionnaires to infer the presence of an eating disorder or muscle dysmorphia without a corresponding clinical interview will be excluded. Likewise, studies that sampled participants based on a self-reported diagnosis and not a formal diagnosis from a relevant medical professional will also be excluded. The presence of comorbidities (e.g., depression, generalised anxiety disorder, or obsessive-compulsive disorder) will not be used to determine inclusion or exclusion.

#### Interventions

Studies that used any type of biopsychosocial treatment and reported longitudinal changes in compulsive exercise will be considered for inclusion in this review. These interventions may consist of, but may not be limited to, cognitive-behavioural therapy, psychoeducation, sport therapy, structured exercise programs, physical exercise and dietary therapy, exposure and response prevention, pharmacotherapy, and multi-component strategies. Both individual and group interventions will be included. We will not place restrictions on the treatment setting, for example, inpatient, outpatient, self-help app, and web-based interventions are eligible for inclusion. There will also be no restrictions on the length of treatment or the number of treatment sessions provided over the course of the intervention.

#### Outcomes of interest

The primary outcome of interest in this review is changes in compulsive exercise from pre- to post-intervention. Although compulsive exercise is the preferred term to describe problematic exercise behaviour in patients with eating disorders, its use did not reach consensus in a recent Delphi study [37]. Thus, a number of other related terms will also be included as primary outcomes, including exercise addiction, exercise dependence, and obligatory exercise (see Appendix for the full list of terms used in the search strategy). We will only include studies that measured compulsive exercise or a related construct using a standardised instrument, such as the Compulsive Exercise Test [49], Exercise Addiction Inventory [17], or Obligatory Exercise Questionnaire [47]. We will exclude studies that only reported on the quantity or frequency of exercise performed without assessing the corresponding qualitative relationship with exercise. Secondary outcomes of interest in this review are changes in weight, eating disorder psychopathology, and other relevant psychological outcomes (e.g., mood and anxiety).

### Search strategy and study selection

The following five electronic databases will be searched from database inception until November 2020: (1) PsycInfo, (2) MEDLINE, (3) Embase, (4) Web of Science, and (5) Scopus. These particular databases were chosen in consultation with an experienced university librarian who specialises in systematic reviews in the psychological sciences. To find additional eligible studies, the reference lists of included articles will be manually searched. Studies citing relevant articles will also be reviewed using the ‘cited by’ function in Google Scholar. We will conduct manual searches for unpublished and ongoing randomised controlled trials in international registers (e.g., www.clinical-trials.gov). If a relevant conference abstract is returned without a corresponding full text article, publications of the lead author will be searched. Papers are required to be peer-reviewed and written in English for inclusion in this review. We did not place restrictions on the date of publication or the geographical location where the research was conducted.

We developed a preliminary search strategy in PsycInfo that was modified in response to suggestions from the aforementioned university librarian. The final search strategy (see Appendix) comprised three clusters of terms relating to: (a) unhealthy exercise behaviour (e.g., compulsive exercise, exercise addiction, and obligatory exercise); (b) diagnostic labels for eating disorders (e.g., anorexia, bulimia, and binge eating disorder) and muscle dysmorphia; and (c) study design (e.g., intervention, treatment, and protocol).

One reviewer (JM) will screen all titles and abstracts against the eligibility criteria after removing duplicates and full texts will be obtained for papers deemed relevant or where inclusion was uncertain. Another reviewer (PA) will cross-review 20% of excluded papers, selected at random. Both reviewers (JM and PA) will review all obtained full texts against the eligibility criteria. Queries regarding eligibility of articles will be resolved through discussion with a third reviewer (SM).

### Data extraction

Data from included articles will be extracted by one author (JM) and checked for accuracy by a second reviewer (PA). The following information will be extracted from each article: (a) study characteristics (title, lead author, year of publication, country, and language); (b) participant characteristics (sample size, age and sex distribution, ethnicity, socioeconomic status, type of disorder, diagnostic classification system, baseline body mass index, comorbidities, duration of illness, dropout rates); (c) study methodology (type of study, study setting, intervention description, comparison group description, length of intervention, and number of treatment sessions); (d) compulsive exercise assessment (questionnaire name, questionnaire domains reported, and assessment time points); and (e) study results (statistical measures used, and primary and secondary outcomes results reported across all time points).

### Quality assessment

The methodological quality of included studies will be appraised using a modified version of the Downs and Black [13] criteria, as amended by Ferro and Speechley [15] to encompass a broad range of study designs. This modified quality checklist excluded items specific to randomised controlled trials, such as those items assessing randomisation, dropouts, blinding, and intervention integrity. The amended quality checklist had 15 items, as opposed to the original 27, and dichotomously scored items as 0 (unable to determine/no) or 1 (yes). The amended checklist assesses four domains: (1) reporting (seven items), (2) external validity (three items), (3) internal validity (four items), and (4) statistical power (one item). Scores from these four domains can be summed to determine an overall quality score for each study. The maximum checklist score for each study is 15 with higher scores (quality score > 10) indicative of greater methodological quality [15].

Two reviewers (JM and PA) will independently score all included studies after data extraction has been completed. Inter-rater agreement will be calculated using the kappa statistic. Large discrepancies in quality evaluation will be resolved through discussion with a third reviewer (SM).

### Data synthesis

Results for this review will be reported using narrative synthesis. Meta-analysis was considered but is likely not feasible taking into account the heterogeneity of study designs, intervention approaches, compulsive exercise instruments, and assessment intervals. Studies receiving a higher overall quality score will be weighted more in the narrative synthesis than studies with a lower overall quality score.

### Dissemination

Findings from this review will be published in a peer-reviewed scientific journal and may be presented by the lead author (JM) at national and international conferences. Data extracted from individual articles will be made available with the full manuscript.

## Discussion

The aim of this systematic review is to summarise the efficacy of existing treatments for compulsive exercise in eating disorders and muscle dysmorphia. Building upon the consensus that targeted interventions are needed to address compulsive exercise in eating disorder populations [37], there has been a rapid upsurge in the number of treatment interventions. It will be useful to determine the most promising treatment components in these interventions to help inform future clinical practice guidelines.

There are a number of strengths of our method. We will include a broad range of samples (adolescents and adults), treatment approaches (e.g., CBT, sport therapy, and multi-component strategies), and study designs (e.g., randomised controlled trials, pilot studies, and case studies) to provide a complete overview of the literature. Secondly, we will conduct a detailed quality assessment of all included studies using an established assessment checklist [13], as amended by Ferro and Speechley [15] to apply to a broad range of study designs. Thirdly, we decided to review treatment interventions for muscle dysmorphia, in addition to eating disorders, as compulsive exercise is a core feature of both groups of disorders and there is a high rate of diagnostic cross-over between these illness groups. Finally, to facilitate transparency, the full search strategy that will be used to find relevant studies has been made available (see Appendix) and all data extracted from included studies will be published with the final manuscript.

The main limitation of this review is the heterogeneity in questionnaires used to assess problematic exercise behaviour. As a consequence, not all included studies will measure compulsive exercise per se, but may measure other related constructs like exercise addiction or exercise dependence. However, considering the theoretical overlap between these constructs, we do not expect substantial differences in results between studies as a function of the choice of exercise behaviour questionnaire. Moreover, due to large variations in treatment approaches, exercise questionnaires, study designs, and assessment intervals, we do not anticipate that a meta-analysis will be feasible. We also anticipate that few, if any, randomised controlled trials will be returned for samples of patients with muscle dysmorphia. Nonetheless, a systematic review of the literature is still warranted to test this supposition and highlight areas for future research in this burgeoning field of clinical psychology.

## Data Availability

The complete PsycInfo search strategy can be found in Appendix. There were no other datasets or materials to disclose.

## Appendix

### PsycInfo Search Strategy

1. (compulsive exercise*).ti,ab,mp
2. (exercise addiction).ti,ab,mp
3. (exercise dependence).ti,ab,mp
4. (excessive exercise*).ti,ab,mp
5. (unhealthy exercise*).ti,ab,mp
6. (obligatory exercise).ti,ab,mp
7. (driven exercise).ti,ab,mp
8. (rigid exercise). ti,ab,mp
9. (exercise abuse).ti,ab,mp
10. (exercise pathology).ti,ab,mp
11. (pathological exercise).ti,ab,mp
12. overexercis*.ti,ab,mp
13. over-exercis*.ti,ab,mp
14. overtraining.ti,ab,mp
15. OTS.ti,ab,mp
16. or/1–15
17. (eating disorder*).ti,ab,mp
18. ED.ti,ab,mp
19. (disordered eating).ti,ab,mp
20. anorexia.ti,ab,mp
21. bulimia.ti,ab,mp
22. (binge eating).ti,ab,mp
23. (eating disorder not otherwise specified).ti,ab,mp
24. EDNOS.ti,ab,mp
25. (other specified feeding or eating disorder).ti,ab,mp
26. OSFED.ti,ab
27. (muscle dysmorphi*).ti,ab,mp
28. bigorexia.ti,ab,mp
29. (reverse anorexia).ti,ab,mp
30. (body dysmorph*).ti,ab,mp
31. exp. eating disorders/
32. exp. anorexia nervosa/
33. exp. bulimia/
34. exp. binge eating disorder/
35. exp. body dysmorphic disorder/
36. or/17–35
37. treatment.ti,ab,mp
38. intervention.ti,ab,mp
39. therap*.ti,ab,mp
40. program*.ti,ab,mp
41. protocol.ti,ab,mp
42. regimen.ti,ab,mp
43. exp. treatment/
44. exp. intervention/
45. or/37–44
46. 16 and 36 and 45
